# Genetic Polymorphisms and Drug Susceptibility in Four Isolates of *Leishmania tropica* Obtained from Canadian Soldiers Returning from Afghanistan

**DOI:** 10.1371/journal.pntd.0001463

**Published:** 2012-01-17

**Authors:** Marie Plourde, Adriano Coelho, Yoav Keynan, Oscar E. Larios, Momar Ndao, Annie Ruest, Gaétan Roy, Ethan Rubinstein, Marc Ouellette

**Affiliations:** 1 Centre de Recherche en Infectiologie du Centre de Recherche du CHUQ and Département de Microbiologie, Immunologie et Infectiologie, Faculté de Médecine, Université Laval, Québec, Québec, Canada; 2 Laboratory of Viral Immunology, Department of Medical Microbiology and Infectious Diseases, University of Manitoba, Winnipeg, Manitoba, Canada; 3 Department of Medicine and Laboratory Medicine, Divisions of Infectious Diseases and Microbiology, University of Saskatchewan, Saskatoon, Saskatchewan, Canada; 4 National Reference Center for Parasitology, McGill University, Montreal General Hospital/Research Institute, Montréal, Québec, Canada; 5 Pavillon Hôtel-Dieu du CHUQ, Québec, Canada; National Institutes of Health, United States of America

## Abstract

**Background:**

Cutaneous leishmaniasis (CL) is a vector-borne parasitic disease characterized by the presence of one or more lesions on the skin that usually heal spontaneously after a few months. Most cases of CL worldwide occur in Southwest Asia, Africa and South America, and a number of cases have been reported among troops deployed to Afghanistan. No vaccines are available against this disease, and its treatment relies on chemotherapy. The aim of this study was to characterize parasites isolated from Canadian soldiers at the molecular level and to determine their susceptibility profile against a panel of antileishmanials to identify appropriate therapies.

**Methodology/Principal Findings:**

Parasites were isolated from skin lesions and characterized as *Leishmania tropica* based on their pulsed field gel electrophoresis profiles and pteridine reductase 1 (PTR1) sequences. Unusually high allelic polymorphisms were observed at several genetic loci for the *L. tropica* isolates that were characterized. The drug susceptibility profile of intracellular amastigote parasites was determined using an established macrophage assay. All isolates were sensitive to miltefosine, amphotericin B, sodium stibogluconate (Pentostam) and paromomycin, but were not susceptible to fluconazole. Variable levels of susceptibility were observed for the antimalarial agent atovaquone/proguanil (Malarone). Three Canadian soldiers from this study were successfully treated with miltefosine.

**Conclusions/Significance:**

This study shows high heterogeneity between the two *L. tropica* allelic versions of a gene but despite this, *L. tropica* isolated from Afghanistan are susceptible to several of the antileishmanial drugs available.

## Author Summary

Cutaneous leishmaniasis (CL) is a vector-borne parasitic disease transmitted by the bite of sandflies, resulting in sores on the skin. No vaccines are available, and treatment relies on chemotherapy. CL has been frequently diagnosed in military personnel deployed to Afghanistan and returning from duty. The parasites isolated from Canadian soldiers were characterized by pulsed field gels and by sequencing conserved genes and were identified as *Leishmania tropica*. In contrast to other Leishmania species, high allelic polymorphisms were observed at several genetic loci for the *L. tropica* isolates that were characterized. *In vitro* susceptibility testing in macrophages showed that all isolates, despite their genetic heterogeneity, were sensitive to most antileishmanial drugs (antimonials, miltefosine, amphotericin B, paromomycin) but were insensitive to fluconazole. This study suggests a number of therapeutic regimens for treating cutaneous leishmaniasis caused by *L. tropica* among patients and soldiers returning from Afghanistan. Canadian soldiers from this study were successfully treated with miltefosine.

## Introduction

Cutaneous leishmaniasis (CL) is a vector-borne parasitic disease characterized by one or more sores or nodules on the skin that often heal spontaneously after a few months, resulting in scar formation. This disease has been frequently diagnosed in military personnel who were returning from duty in Southwest Asia [1], [2], with several outbreaks observed in troops deployed to Iraq [3] and Afghanistan [2], [4]. Currently, Kabul is believed to be the largest focus of CL worldwide, having an estimated incidence of 67,500 new cases per annum [5]. Whereas CL in Iraq has been mostly caused by *Leishmania major*, CL in Afghanistan can either be due to *Leishmania tropica* or *Leishmania major* [6], and differences in clinical features have been observed between the two species. Notably, *L. tropica* tends to cause more chronic infections and may rarely progress to a systemic form of the disease termed viscerotropic leishmaniasis, a situation requiring special attention [7].

There is a lack of consensus about the best therapeutic options for the treatment of CL, mainly due to the lack of properly controlled clinical trials [8]. Because of the self-healing nature of the illness, the treatment of CL depends on several factors such as the site and number of lesions, the aetiology of the disease, and personal preferences. One of the main therapeutic options that has been used for the treatment of CL for many years relied on the local or systemic administration of pentavalent antimony [9]. Because *Leishmania* species are susceptible to heat, the local application of radio frequency to generate heat at the site of the lesions was also shown to yield cure rates equivalent to systemic pentavalent antimony [10], [11]. Nonetheless, the availability of effective oral treatments would constitute attractive therapeutic options against CL, and there is evidence of benefit for the use of oral triazoles like itraconazole and fluconazole against *L. tropica* and *L. major*, respectively [8]. Miltefosine, another orally administered drug, was shown to be an effective treatment against visceral leishmaniasis in India [12] and cutaneous leishmaniasis in South America [13], but there is only limited data about its efficacy against CL in Southwest Asia [14]–[16].

In this report, we describe the molecular characterization and *in vitro* drug susceptibility profiles of *Leishmania* parasites isolated from four Canadian soldiers suffering from CL after returning from Afghanistan. Primary treatment based on oral fluconazole failed to improve the appearance of lesions in three of them. We show that *L. tropica* was responsible for the lesions in every patient and that the parasites are highly heterogeneous but nonetheless remained sensitive to most known antileishmanials.

## Methods

### Ethics Statement

The skin biopsies were taken after appropriate informed consent was obtained, and as part of the routine patient care. Leishmania parasite isolates were submitted for susceptibility testing in order to assist in the clinical management of individuals with suboptimal response to fluconazole. No additional samples or procedures were done.

### Parasites and culture

Fresh tissue samples obtained through biopsy of the skin lesions were collected from three Canadian soldiers who returned from duty in southern Afghanistan with suspected CL lesions at the Department of Medical Microbiology and Infectious Diseases of the University of Manitoba in Winnipeg. Samples were submitted to culture, pathological examination, and PCR analyses. The histology revealed the presence of granulotomatous inflammation. The isolates identified as 017102, 431462, and 072218 underwent routine clinical laboratory studies at the National Reference Center for Parasitology in Montreal, QC. An additional skin lesion sample (identified as 18693) was collected from a Canadian soldier also returning from Afghanistan and suspected of suffering from CL at the CHUQ in Quebec, QC. Parasites were isolated from the biopsy in SDM-79 medium supplemented with 20% heat-inactivated fetal calf serum, 5 µg/ml hemin and 10 µM biopterin at pH 7.0 and 25°C. The molecular characterization of parasites was done at the Centre de Recherche en Infectiologie du Centre de Recherche du CHUL, Quebec, QC. The *L. tropica* strains 175 and 482, isolated from Iranian patients [17], and *L. tropica* MHOM/SU/74/K27, obtained from the ATCC, were used as reference isolates.

### Pulsed field gel electrophoresis karyotyping

Agarose blocks containing *Leishmania* cells were prepared as described [18]. Briefly, cells were resuspended in HEPES buffer at a density of 5×10^8^ cells/ml and mixed with low-melting-point agarose. Cells were lysed in the presence of 0.5 M EDTA (pH 9.5), 1% SLS, and proteinase K (500 µg/ml). Their chromosomes were electrophoresed by a BioRad (Hercules, California, United States) contour-clamped homogeneous electric field (CHEF) mapper for separating 0.1–1.0 Mbp DNAs over a period of 27 h. Chromosomes were visualized after ethidium bromide staining.

### Sequence analysis

Species identification and heterogeneity were studied by sequencing the pteridine reductase 1 (*PTR1*), glucose-6-phosphate isomerase (*GPI*), nucleoside hydrolase 1 (*NH1*), dihydrofolate reductase-thymidylate synthase (*DHFR-TS*), stearic acid desaturase (*SAD*), mannose phosphate isomerase (*MPI*), aspartate aminotransferase (*ASAT*), 6-phosphogluconate dehydrogenase (*PGD*), glucose-6-phosphate dehydrogenase (*G6PDH*) and cytochrome B (*CYTB*) genes. Genomic DNA was extracted from mid-log phase parasites using the DNAzol reagent (Invitrogen) as described by the manufacturer. PCR reactions were performed in 50 µl using the primers listed in Table S1 and contained 100 ng of total gDNA, 50 pmol of each primer, 0.2 mM of dNTPs, 1.5 mM of MgCl2 and 5 U of Taq polymerase. Amplification was performed in 30 cycles, each cycle using the following conditions: denaturation at 94°C for 1 min, annealing at 58°C for 1 min and extension at 72°C for 1–2 min (depending on the size of PCR products). A final extension was performed at 72°C for 5 min. PCR products were migrated on agarose gel, purified with the QIAquick Gel Extraction Kit (Qiagen) and sequenced with an ABI Prism 3100 DNA sequencer. The *PTR1* sequences obtained were compared to those of eight *Leishmania* reference isolates using the Lasergene Software (DNASTAR, Inc.).

### Phylogenetic analysis

Multiple sequence alignments were performed on the amino acid sequence of the *PTR1* coding region using ClustalW [19] with the default settings. The resulting multiple alignments were subjected to phylogenetic analysis using the neighbor-joining method [20] with the Poisson correction distance method as implemented in the MEGA3.1 software [21]. The reliabilities of each branch point were assessed by the analysis of 1000 bootstrap replicates.

### Viability test

The 50% inhibitory concentrations (IC_50_) of drugs on macrophages were established by using the 3-(4,5-dimethylthiazol-2-yl)-2,5-diphenyltetrazolium bromide (MTT) assay. Briefly, THP-1 cells were differentiated in 96-well flat-bottom microtiter plates in a volume of 100 µl of complete RPMI 1640 medium supplemented with 10% heat-inactivated fetal calf serum and 20 ng/ml phorbol myristate acetate. Plates were incubated at 37°C in the presence of 5% CO_2_ for 3 days. Drugs were added at 1/10 of the final concentration in a volume of 10 µl in duplicate. After 96 h of incubation, 10 µl of MTT (10 mg/ml) was added to each well and plates were further incubated for 4 h. The enzymatic reaction was stopped by the addition of 100 µl of 50% ethanol-10% sodium dodecyl sulfate. The plates were incubated for an additional 30 minutes under agitation at room temperature before reading the optical density at 570 nm with a 96-well scanner. The viability assays were performed in duplicates. As a control, the cytotoxicity of reagents used to solubilize the drugs was determined and no substantial toxicity was found.

### Drug susceptibility assays

*L. tropica* promastigote parasites were transfected with the firefly luciferase-containing vector pSP1.2 LUC αHYGα as previously described [22]. THP-1 cells were differentiated by incubation at 37°C in the presence of 5% CO_2_ for 3 days in complete RPMI 1640 medium supplemented with 10% heat-inactivated fetal calf serum and 20 ng/ml phorbol myristate acetate. The cells were washed with pre-warmed medium and subsequently infected with *L. tropica* promastigotes at a parasite/macrophage ratio of 15∶1 for 3 h. Non-internalized parasites were removed by several washes. Luciferase activity was measured after 4 days of exposure to fluconazole, Pentostam, amphotericin B, miltefosine, paromomycin or Malarone as described elsewhere [23].

## Results

### Molecular characterization of *Leishmania* isolates

**Figure 1:**
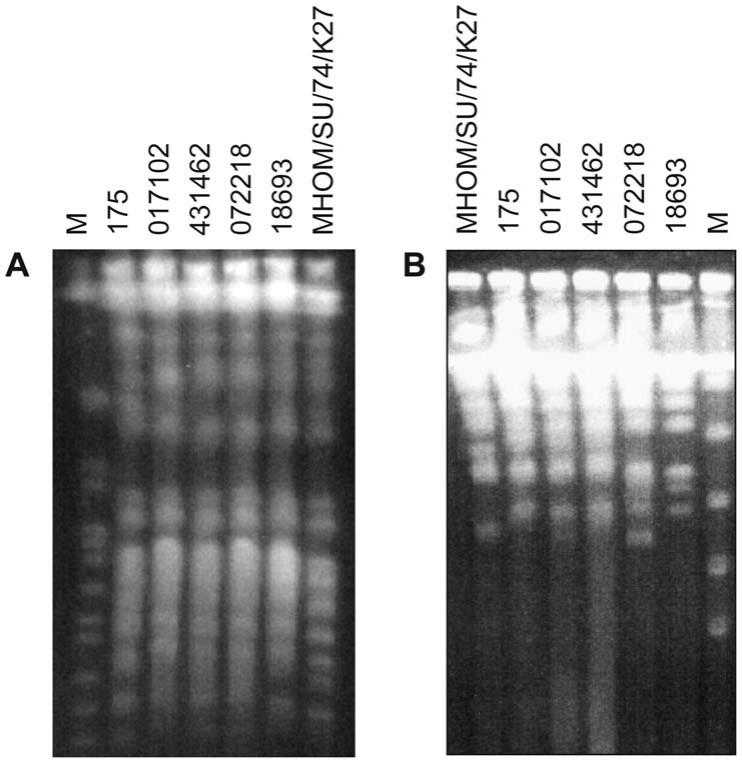
Karyotypes of Afghan and Iranian *Leishmania tropica* isolates as characterized by PFGE. Cells were embedded and lyzed in agarose and their chromosomes were electrophoresed and stained with ethidium bromide. **A.** 600–1300 kb electrophoresis **B.** 100–500 kb electrophoresis. The field isolates 017102, 431462, 072218 and 18693 from Afghanistan have closely related karyotype to the *L. tropica* reference strains 175 and MHOM/SU/74/K27. M, yeast chromosomes molecular weight marker (BioRad).

Parasites recovered from biopsy samples of four Canadian military personnel who returned from deployment in Kandahar, Afghanistan, with clinical manifestations of CL were characterized by pulsed field gel electrophoresis (PFGE). PFGE conditions optimized for the analysis of larger chromosomes did not show any major differences in the chromosome numbers and sizes between our isolates (Fig. 1A) and revealed that they were genetically closely related to the ATCC *L. tropica* strain MHOM/SU/74/K27 and to a *L. tropica* isolate recovered from a patient suffering from CL in Iran (*L. tropica* 175) [17], [24]. The analysis of smaller chromosomes revealed considerable karyotype differences, however (Fig. 1B).

**Figure 2:**
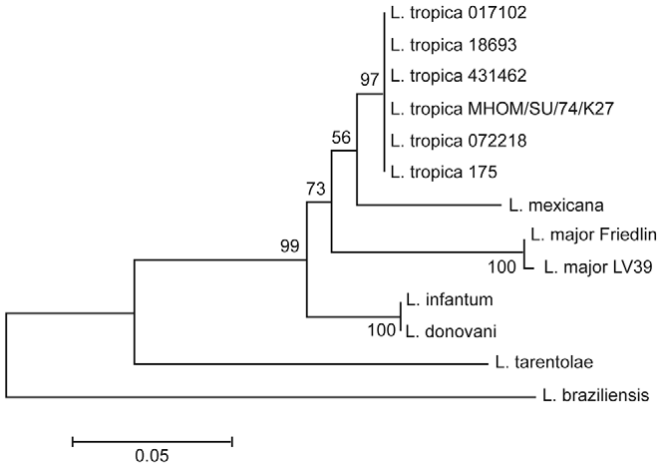
Phylogenetic analysis of the PTR1 sequences. Amino acid sequences were aligned using the ClustalW algorithm. The resulting multiple alignment was subjected to phylogenetic analysis by using the neighbor-joining method with Poisson correction as implemented in the MEGA3.1 software. The field isolates 017102, 431462, 072218, and 18693 from Afghanistan are clustering with the *L. tropica* reference strains 175 and MHOM/SU/74/K27. The reliabilities of each branch point were assessed by the analysis of 1000 bootstrap replicates.

The isolates were further characterized on the basis of the pteridine reductase 1 (PTR1) sequence [17]. PCR fragments of the coding region of *PTR1* were amplified from genomic DNA extracted from the clinical isolates and sequenced. The sequences generated were compared to those of eight *Leishmania* reference strains and were shown to be closely related to *L. tropica* sequences (data not shown). A neighbor-joining phylogenetic analysis generated from the translated *PTR1* sequences further confirmed that the four CL strains derived from Canadian soldiers were *L. tropica* parasites (Fig. 2).

### *Leishmania tropica* genetic heterozygosity

The *PTR1* nucleotide sequences of the four *L. tropica* isolated from Canadian soldiers revealed the presence of single nucleotide polymorphisms (SNPs) at five different positions (Table 1). The changes in nucleotide were conservative (Table 1), and the same polymorphisms were also observed in two other strains of *L. tropica* (strains 482 and MHOM/SU/74/K27) that we have analyzed (Table 2). The heterozygous gene sequences were detected as split peaks in the chromatogram generated by the sequencing of the *PTR1* locus in both directions, using DNA extracted from populations of parasites. This type of polymorphism was not observed when sequencing the *PTR1* gene of *L. infantum* and *L. major* (Table 2). To assess whether these polymorphisms corresponded to population heterogeneity or to parasite heterozygocity, the *PTR1* sequence of cloned parasites from three distinct *L. tropica* strains (MHOM/SU/74/K27, 072218 and 482) was determined (3 clones for each strain). Again, the same *PTR1* polymorphisms were detected in every clone tested. Each allele was detected at a frequency of 50%, which suggests that parasites were harbouring two distinct alleles. The same two alleles were detected in every *L. tropica* strain studied (Table 1), which is reflected by the homogenous clustering of the *L. tropica* isolates in the neighbour-joining phylogenetic analysis (Fig. 2).

**Table 1:** Polymorphisms in *L. tropica* isolates^a^^,^ b.

Genes^c^	Nucleotides^d^	Amino acids
*PTR1*	G 243 A	Ala 81 Ala
	G 561 T	Pro 187 Pro
	T 624 A	Ala 208 Ala
	A 642 G	Pro 214 Pro
	T 690 C	Ala 230 Ala
*NH1*	T 243 A	Ile 81 Ile
	A 698 G	His 233 Arg
	**C 752 G**	**Pro 251 Arg**
	G 828 T	Ala 276 Ala
*DHFRTS*	**A 911 G**	**Gln 304 Arg**
	G 1095 A	Gln 365 Gln
	A 1149 G	Leu 383 Leu
	**A 1469 C**	**Glu 490 Ala**
*SAD*	C 189 T	Ser 63 Ser
	C 1251 T	Gly 417 Gly
	**T 1335 A**	**Asp 445 Glu**
*GPI*	**G 109 T**	**Ala 37 Ser**
	C 135 G	Ala 45 Ala
	**G 230 A**	**Ser 77 Asn**
	**C 383 T**	**Ala 128 Val**
	C 699 T	Asn 233 Asn
	C 888 T	Val 296 Val
	T 1290 C	Ile 430 Ile
*PGD*	G 54 C	Ala 18 Ala
	A 114 G	Thr 38 Thr
	G 249 T	Thr 83 Thr
	A 447 G	Pro 149 Pro
	A 489 G	Ala 163 Ala
	**G 623 T**	**Arg 208 Leu**
	**G 666 T**	**Glu 222 Asp**
	**A 679 G**	**Asn 227 Asp**
	**G 876 C**	**Met 292 Ile**
	**A 884 G**	**Tyr 295 Cys**
	G 903 C	Ala 301 Ala
	G 1056 C	Leu 352 Leu
	**A 1228 G**	**Asn 410 Lys**
	C1293 T	Ala 431 Ala
	G 1383 C	Gly 461 Gly
*ASAT*	**A 55 C**	**Lys 19 Gln**
	A 978 C	Ala 326 Ala
	**C 1141 T**	**Pro 381 Ser**
*G6PDH*	**A 178 G**	**Asn 60 Asp**
	A 198 A	Glu 66 Glu
	C 294 T	Gly 98 Gly
	**A 463 G**	**Asn 155 Asp**
	T 669 C	Gly 223 Gly
	A 1038 G	Pro 346 Pro
	**A 1039 G**	**Ile 347 Val**
	G 1083 C	Ala 361 Ala
	C 1191 T	Gly 397 Gly
	A 1542 G	Pro 514 Pro

a Nucleotide polymorphisms and corresponding amino acid changes are shown.

b The polymorphisms in bold cause an amino acid substitution.

c The polymorphisms indicated were common to every *L. tropica* isolates analyzed, except for *PGD*, *G6PDH*, and *SAD*, which were not polymorphic in *L. tropica* MHOM/SU/74/K27.

d For nucleotide numbering, +1 corresponds to the A of the ATG translation initiation codon.

*PTR1* – pteridine reductase 1; *NH1* – nucleoside hydrolase 1; *DHFRTS* – dihydrofolate reductase-thymidylate synthase; *SAD* – stearic acid desaturase; *GPI* – glucose-6-phosphate isomerase; *PGD* – 6-phosphogluconate dehydrogenase; *ASAT* – aspartate aminotransferase; *G6PDH* – glucose-6-phosphate dehydrogenase.

**Table 2:** Single nucleotide polymorphisms within *Leishmania* species for 10 genes^a^.

Gene	*GPI*	*PTR1*	*NH1*	*PGD*	*ASAT*	*G6PDH*	*DHFR-TS*	*SAD*	*MPI*	*CYTB*
Chromosome	12	23	29	35	24	34	6	14	32	*
*L. major*Friedlin	0	0	0	0	0	0	0	0	0	0
*L. infantum*JPCM5	0	0	0	0	0	0	0	0	0	0
*L. infantum*LEM 3843	0	0	0	ND	ND	ND	ND	ND	ND	ND
*L. donovani infantum*	0	0	0	ND	ND	ND	ND	ND	ND	ND
*L. tropica*MHOM/SU/74/K27	7	5	4	0	3	0	4	0	0	0
*L. tropica*IRAN 482	7	5	4	15	6	10	4	3	0	0
*L. tropica*072218	7	5	4	15	6	10	4	3	0	0

ND – not done.

a For each gene, the number of heterozygous sites is indicated.

*: The *CYTB* gene is located on kinetoplast DNA.

Abreviations: *PTR1* – pteridine reductase 1; *NH1* – nucleoside hydrolase 1; *DHFR-TS* – dihydrofolate reductase-thymidylate synthase; *SAD* – stearic acid desaturase; *GPI* – glucose-6-phosphate isomerise; *PGD* – 6-phosphogluconate dehydrogenase; *ASAT* – aspartate aminotransferase; *G6PDH* – glucose-6-phosphate dehydrogenase; *MPI* – mannose phosphate isomerise; *CYTB* – Cytochrome B.

To assess the extent of the genetic polymorphism in our panel of *L. tropica* strains, nine additional genes located on distinct chromosomes, i.e. glucose-6-phosphate isomerase (*GPI*), nucleoside hydrolase 1 (*NH1*), dihydrofolate reductase-thymidylate synthase (*DHFR-TS*), stearic acid desaturase (*SAD*), mannose phosphate isomerase (*MPI*), aspartate aminotransferase (*ASAT*), 6-phosphogluconate dehydrogenase (*PGD*), glucose-6-phosphate dehydrogenase (*G6PDH*), and cytochrome B (*CYTB*), were also sequenced in clones of strains 482, 072218 and MHOM/SU/74/K27. Heterozygous sites were observed at every locus (Table 1) except for *MPI* and *CYTB* (Table 2). In addition, the same alleles were detected in every *L. tropica* strains studied, except for the *ASAT* gene, which had three additional polymorphic sites common to *L. tropica* 482 and 072218 that were absent from *L. tropica* MHOM/SU/74/K27 (Table 2), and the *PGD*, *G6PDH*, and *SAD* genes, which were not polymorphic in *L. tropica* MHOM/SU/74/K27 (Table 2). In contrast to the *PTR1* locus, however, the polymorphisms observed at the *NH1*, *DHFR-TS*, *SAD*, *PGD*, *G6PDH*, *ASAT*, and *GPI* loci were non-conservative for at least one position, with some genes having several non-conservative heterozygous sites (Table 1). These polymorphisms were not observed in other species outside *L. tropica* (Table 2). To rule out polymerase errors, the sequencing of at least three independent PCR products was done for each particular nucleotide position, as it would be quite rare that the presence of the same SNPs in different PCR reaction comes from polymerase errors. Moreover, for some genes (for example PTR1), PCR products were TA cloned and independent clones were sequenced for the confirmation of the SNPs using primers within the pGEM-T easy vector, hence ruling out the possibility of polymerase errors.

### Drug susceptibility profiles of *L. tropica* clinical isolates

**Figure 3:**
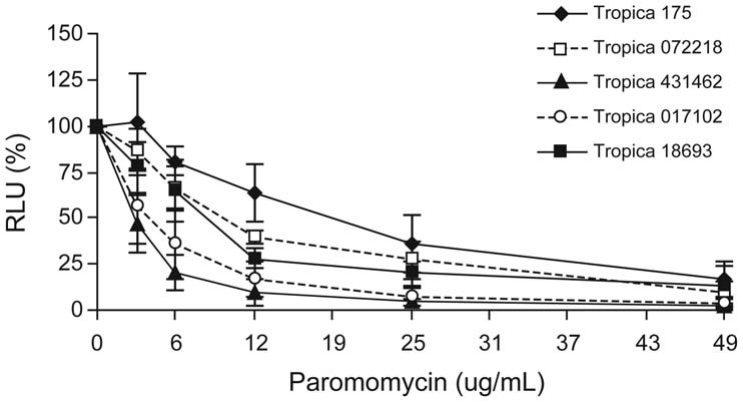
Paromomycin susceptibility of *Leishmania tropica* field isolates recovered from soldiers suffering from CL. The four *L. tropica* isolates from Afghanistan are sensitive to paromomycin as intracellular amastigotes. Intracellular parasites were incubated for 4 days with the indicated concentrations of paromomycin. The mean of two independent experiments done in duplicate is shown. Values are represented as numbers of relative light units (RLU).

Three of the four Canadian soldiers were initially treated with oral fluconazole without any clinical improvements. To establish whether the therapeutic failure was due to parasites unresponsive to fluconazole and to test whether these parasites were sensitive to classical antileishmanials, *in vitro* susceptibility testing was performed with the four *L. tropica* isolates using the human monocyte cell line THP-1 and recombinant parasites transfected with the firefly luciferase gene. The latter system is a convenient and rapid quantitative method to monitor the growth of intracellular parasites [23]. Growth was compared between mock-treated parasites and parasites exposed to fluconazole, Pentostam, amphotericin B, miltefosine, and paromomycin. We observed that *L. tropica* intracellular amastigotes were insensitive to fluconazole at concentrations that were achievable *in vitro* (Table 3). Fluconazole was highly active when we tested it against *C. albicans* (results not shown). In contrast, when compared to our *L. tropica* reference strain 175, all *L. tropica* isolates tested were sensitive to amphotericin B, miltefosine, Pentostam, and paromomycin as intracellular parasites (Table 3 and Figure 3). The only exception was *L. tropica* 18693, for which a slightly higher miltefosine EC_50_ was observed in comparison to the other *L. tropica* strains (Table 3).

**Table 3:** Susceptibility of *L. tropica* clinical isolates to antileishmanial drugs.

Strains			EC_50_ a			
	AmphotericinB (µg/ml)	Paromomycin (µg/ml)	Miltefosine (µg/ml)	Pentostam (µg/ml)	Malarone (µg/ml)	Fluconazole(µg/ml)
175	0.51±0.08	19.0±8.1	0.19±0.07	300±32	>8	>275
017102	0.59±0.29	7.4±1.7	0.11±0.004	188±39	6.6±0.1	>275
431462	0.32±0.18	3.2±0.9	0.09±0.004	142±4	3.6±0.6	>275
072218	0.51±0.07	9.8±1.8	0.15±0.07	142±12	4.4±1.1	>275
18693	0.72±0.33	5.8±4.8	0.65±0.33	ND	>8	>275

a Determined as amastigotes in THP-1 cells.

One soldier infected by CL had to travel to Central Africa and was on Malarone anti-malaria prophylaxis. Intriguingly, his cutaneous lesions cicatrized while being on Malarone prophylaxis, so we tested whether Malarone had any activity against *L. tropica* isolates using the intracellular amastigote assay. The toxicity of Malarone to the THP-1 cells was first established by MTT viability assay, and this cell line was found to display a Malarone IC_50_ of 32 ug/ml. No THP-1 cytotoxicity was observed for Malarone concentrations up to 10 ug/ml. Using drug concentrations below 10 ug/ml, we found that three *L. tropica* strains were sensitive to Malarone as intracellular amastigotes (Table 3), including the strain that was isolated from the patient whose CL regressed during Malarone prophylaxis.

## Discussion

We describe here the drug susceptibility and molecular characterization of *L. tropica* isolates derived from Canadian soldiers returning from Afghanistan. The isolates were identified as *L. tropica* by phylogenetic studies based on the PTR1 sequence, an approach proven to be useful for the molecular identification of *Leishmania* species [17]. Moreover, the PFGE karyotypes of the recovered *Leishmania* parasites were similar to those of *L. tropica* reference strains. This is consistent with epidemiological data that showed the majority of CL cases in Afghanistan being due to this species [5], [25]. Interestingly, the sequence of *PTR1* revealed several SNPs in distinct *L. tropica* isolates. This phenomenon appeared to be widespread across the *L. tropica* genome, as it was also observed at other genetic loci on different chromosomes. Most of the loci analyzed code for proteins that are part of the panel of enzymes used for the characterization of *Leishmania* species by multilocus enzyme electrophoresis [26]. Among these, six (*GPI*, *NH1*, *ASAT*, *G6PDH*, *PGD*, and *MPI*) were further shown to be useful markers for the molecular characterization of *Leishmania* strains and species [27]–[29]. *CYTB* was chosen as a mitochondrial gene representative, since it has also been reported to be phylogenetically informative [30], [31]. The *SAD* and *DHFR-TS* loci were randomly chosen. DNA sequencing of cloned parasites revealed a number of heterozygous sites at these loci, some of which led to non-conservative changes. Although the prevailing mode of reproduction of *Leishmania* appears to be clonal [32], heterozygosity at several sites within genes or at distinct loci is suggestive of genetic exchange between strains [27], and this phenomenon has previously been observed in other *Leishmania* species [27], [28],[32]–[35]. Most of these studies used microsatellite markers with high mutation rates as indicators of heterozygocity, however, and this is the first report about extensive heterozygocity within coding regions in *L. tropica*. Nonetheless, the heterozygosity of the *L. tropica* isolates appears to be fixed, the same alleles being found among strains for most of the loci studied except for the reference *L. tropica* MHOM/SU/74/K27. This is suggestive of clonal propagation within foci of endemicity and is consistent with the anthroponotic mode of transmission of *L. tropica* in urban and peri-urban environments of Afghanistan [5]. *L. tropica* parasites were known to display genetic heterogeneity at the population level [34]–[37] and to be responsible for a spectrum of clinical manifestations including cutaneous, chronic, or viscerotropic leishmaniasis [7]. Unfortunately, the small number of isolates available for analysis prevented correlating heterozygocity with clinical data or drug susceptibility. However, this seems to be unique to *L. tropica* since other species did not show this level of allelic polymorphism (Table 2).

Although CL is generally self-limiting, the complexity of the clinical spectrum associated with *L. tropica* infections emphasizes the need for treatment. Evidence suggested that the disruption of ergosterol biosynthesis by oral azoles is an effective treatment against CL [38], [39]. However, a species-specific effect was found to be important to the clinical outcome conferred by azole molecules, with itraconazole and fluconazole being more active against *L. tropica* and *L. major*, respectively [8]. Here, the failure of oral fluconazole to improve the appearance of cutaneous lesions was indeed explained by the intrinsic resistance of our *L. tropica* isolates, the amastigote parasites being insensitive to the highest fluconazole concentration achievable *in vitro* using an established intracellular assay (Table 3). All isolates were sensitive to the other drugs tested, however, with the exception of Malarone, for which variable levels of susceptibility were observed. While anecdotal, CL regressed in one soldier during Malarone prophylaxis. Although we cannot exclude spontaneous healing, it might be worthwhile to evaluate the usefulness of this drug against CL in properly controlled experiments.

Miltefosine is an orally administered antileishmanial approved for the treatment of visceral leishmaniasis in India [12], with demonstrated efficacy against CL in some regions of South America [13]. In contrast, mostly sporadic data have been reported regarding the efficacy of miltefosine against CL in Southwest Asia [14]–[16]. Based on the results of our drug susceptibility screening, soldiers were treated with miltefosine and healing of their CL lesions was observed [40]. The patient treated with Malarone received miltefosine but elected to discontinue therapy due to abdominal pain and in the face of a contracting lesion. The other soldiers tolerated medication well and lesions resolved at follow up.

## Supporting Information

Table S1 **Primers used in this study.**
(DOC)
