# Supplementary material for: Genetic Polymorphisms and Drug Susceptibility in Four Isolates of Leishmania tropica Obtained from Canadian Soldiers Returning from Afghanistan
Source: PLoS Negl Trop Dis. 2012 Jan 17;6(1):e1463. doi: 10.1371/journal.pntd.0001463 (PMC3260320; doi:10.1371/journal.pntd.0001463)
Supplement: Table S1 — Primers used in this study. (DOC) [file pntd.0001463.s001.doc]

**Supporting information**

**Table S1.** Primers used in this study.

| GPIF | 5’ - GAA TCC CTT TTC AAG ATG AGC GAT TAT - 3’ |
| --- | --- |
| GPIR | 5’ - CCC CTG AGA GGC AAT CAC AGC - 3’ |
| PTR1F | 5’ - GGC GCC GCG AAG CGT CTT GG - 3’ |
| PTR1R | 5’ - CGC TGG TAG AGG GGC ACC TT - 3’ |
| NH1F | 5’ - GGG ATC GAT GAT GCC GTG GC - 3’ |
| NH1R | 5’ - GAT GCG CTC GAG TGC GTC AA - 3’ |
| DHFRTS F | 5’ - CGC TGT ACT GCA GCT CCA TC - 3’ |
| DHFRTS R | 5’ - CCT ATA CGG CCA TCT CCA TC - 3’ |
| SADF | 5’ - CTG CAG GCC GTG CAG AAC CTC - 3' |
| SADR | 5’ - GGG AAC GTC ACA TCG CCG AGG - 3' |
| MPIF | 5’ - CTA CCT GTC GCT CAA GTC -3’ |
| MPIR | 5’ - ATG TCT GAG CTC GTA AAG CT - 3' |
| CyBF | 5’ - GGT GTA GGT TTT AGT YTA GG -3’ |
| CyBR | 5’ - CTA CAA TAA ACA AAT CAT AAT ATR CAA TT -3’ |

The primers used for the genes *mpi, gpi, nh1* and *cytb* have previously been described [26,27,41].
